# Alleviation of Associated Drought and Salinity Stress’ Detrimental Impacts on an Eggplant Cultivar (‘Bonica F1’) by Adding Biochar

**DOI:** 10.3390/plants12061399

**Published:** 2023-03-21

**Authors:** Sami Hannachi, Angelo Signore, Lassaad Mechi

**Affiliations:** 1 Department of Biology, College of Science, University of Hail, P.O. Box 2440, Ha’il 81451, Saudi Arabia; 2 Department of Plants and Crops, Faculty of Bioscience Engineering, Ghent University, Coupure Links 653, 9000 Ghent, Belgium; 3 Department of Agricultural and Environmental Science, University of Bari Aldo Moro, Via Amendola 165/A, 70126 Bari, Italy; 4 Department of Chemistry, College of Science, University of Hail, P.O. Box 2440, Ha’il 81451, Saudi Arabia

**Keywords:** salt, drought, biochar, *Solanum melongena* L.

## Abstract

To investigate the impact of biochar on eggplant growth, physiology, and yield parameters under separate and associated drought and salt stress, a pot experiment was carried out. An eggplant variety (‘Bonica F1’) was exposed to one NaCl concentration (S1 = 300 mM), three irrigation regimes (FI: full irrigation; DI: deficit irrigation; ARD: alternate root-zone drying irrigation), and one dose of biochar (B1 = 6% by weight). Our findings demonstrated that associated drought and salt stress had a greater negative impact on ‘Bonica F1’ performance in comparison to single drought or salt stress. Whereas, adding biochar to the soil improved the ability of ‘Bonica F1’ to alleviate the single and associated effects of salt and drought stress. Moreover, in comparison to DI under salinity, biochar addition in ARD significantly increased plant height, aerial biomass, fruit number per plant, and mean fresh weight per fruit by 18.4%, 39.7%, 37.5%, and 36.3%, respectively. Furthermore, under limited and saline irrigation, photosynthetic rate (A_n_), transpiration rate (E), and stomatal conductance (g_s_) declined. In addition, the interaction between ARD and biochar effectively restored the equilibrium between the plant chemical signal (ABA) and hydraulic signal (leaf water potential). As a result, mainly under salt stress, with ARD treatment, intrinsic water use efficiency (WUE_i_) and yield traits were much higher than those in DI. Overall, biochar in combination with ARD could be an efficient approach for preserving crop productivity.

## 1. Introduction

Plant growth and development are susceptible to various stresses because of the effects of global warming, including biotic (pathogen infection) and abiotic (extreme temperatures, drought, salt, etc.) factors [1]. Among the abiotic stresses, drought is one of the most important for plants, as it has a detrimental impact on crop yields, quality, and productivity, thus compromising global food security [2,3,4,5]. Abiotic stresses are thought to be responsible for over 80% of crop production losses worldwide, with drought stress accounting for the majority of these losses. Due to anticipated increases in the frequency of extreme climatic events, it has been predicted that droughts will become more frequent and severe around the world in the future [6]. Intrinsic water use efficiency (WUE_i_), meanwhile, is a crucial characteristic for signaling plant tolerance to drought stress [7]. Osmotic pressure caused by drought stress in plants negatively affects stomata closure, CO_2_ fixation, photorespiration, and other plant functions, which would reduce agricultural output [8]. Additionally, drought stress may alter plant phenology (e.g., accelerate or postpone flowering time), which in turn may have an impact on agricultural productivity [9]. According to previous reports, chickpea (*Cicer arietinum* L.) productivity was significantly impacted by a lack of water during the flowering stage [10]. During drought stress, plants always have deeper roots as a mechanism that allows them to absorb a greater amount of water and nutrients from the soil [11]. Another important limiting factor for sustainable agriculture is salinity, which globally suppresses plant development and production [12,13]. Over 7% of the world’s cultivated superficies are under salt stress, and more than 70 countries have been identified as having significant salinity-affected land areas [14]. In addition to altering the physiological development of leaves, salinity stress may also make it more difficult for plant roots to achieve water and mineral uptake (such as N) from the soil [15,16]. The application of biochar to low-fertility soils is a potential strategy to enhance soil fertility and subsequently plant yield [9]. There were fewer studies about the use of biochar with respect to alkaline soil in conditions of drought, but biochar application might boost plant yield specifically in soils with acidic and neutral pH [17,18]. Additionally, biochar has a significant concentration of inorganic carbonates and minerals such as calcium and magnesium that are crucial for plant growth. The permeability, carbon sequestration, and productivity of soils may all be enhanced by biochar [19]. Ref. [20] emphasized the ability of biochar to increase plant productivity by promoting microbial potential in the rhizosphere and enhancing the WHC (water holding capacity) in the soil. In addition, the high biochar porous structure hugely increased soil surface area, improving CEC (the capacity for exchanging cations) [21]. Therefore, large quantities of minerals might be kept in the soil, improving the effectiveness of nutrient uptake and raising plant productivity [22]. Earlier research revealed that biochar’s high porosity allows it to boost soil moisture content and lessen salt stress [23]. Eggplant (*Solanum melongena* L.) is considered one of the most important vegetable crops with high economic value worldwide [24]. China and India are two of the top five countries that produce eggplant [25]. However, several abiotic stresses, including salinity and drought, can have a major detrimental effect on the growth and production of this vital legume crop. The productivity of eggplant, as influenced by salinity and drought, has been extensively studied in the past, but it is still unclear what physiological factors contribute to the decreased yield. Additionally, there is no information on whether adding biochar could be a useful management strategy for soils with low-fertility under the combined salt and drought stresses. Therefore, it will be advantageous for sustainable agriculture to have better knowledge of the physiological benefits of biochar addition and root characteristics for eggplant development under drought and salt stress. This study investigated the effects of biochar on physiological parameters of eggplant leaves, crop production, and WUE_i_ at both leaf and yield levels under the dual stress of salinity and drought. This study’s objective was to assess the single and associated impacts of drought and salinity stress on eggplant productivity. Three primary hypotheses were investigated in this study: (i) Drought stress might affect physiological parameters in leaves and, consequently, crop productivity; (ii) salt stress might exacerbate the impacts of drought stress; and (iii) adding biochar might lessen the impact of both salt and drought and stresses on crop productivity.

## 2. Results

### 2.1. Plant Phenology, Growth, and Yield Properties

Drought stress, salinity stress, and biochar addition greatly impacted eggplant phenology, growth, and Yield properties (Table 1). Biochar dose (B), irrigation regime (I), and NaCl concentration (S) all had a substantial impact on flowering time (*p* = 0.0018; *p* = 0.0031; *p* = 0.0028), plant height (*p* = 0.0021; *p* = 0.0022; *p* = 0.0025), aerial biomass (*p* = 0.0019; *p* = 0.0021; *p* = 0.0018), fruit per plant (*p* = 0.0013; *p* = 0.0021; *p* = 0.0035) and mean fresh weight per fruit (*p* = 0.0012; *p* = 0.0019; *p* = 0.0013) (Table 1). S + I greatly affected plant height (*p* = 0.0017), while B + S and S + I significantly affected fruit per plant (*p* = 0.0021; *p* = 0.0029) and mean fresh weight per fruit (*p* = 0.035; *p* = 0.0022). Interactions had no effect on the aerial biomass. Higher plant height, aerial biomass, fruit per plant, and mean fresh weight per fruit were all seen in combined FI (full irrigation) and biochar amendment, and these values were all considerably greater than in DI (deficit irrigation) (*p* = 0.014; *p* = 0.025; *p* = 0.045; *p* = 0.025) and associated ARD (alternate root-zone drying irrigation) (*p* = 0.023; *p* = 0.017; *p* = 0.052; *p* = 0.031) and biochar addition in presence or absence of salt stress condition. Moreover, adding biochar to the soil increased plant height (PH), aerial biomass (AB), fruit per plant (N), and mean fresh weight per fruit (FW) by 18.4%, 39.7%, 37.5%, and 36.3%, respectively, in combination with ARD and salt stress (300 mM NaCl), compared with the association of DI and S (300 mM of NaCl).

### 2.2. Physiological Parameters

**Figure 1:**
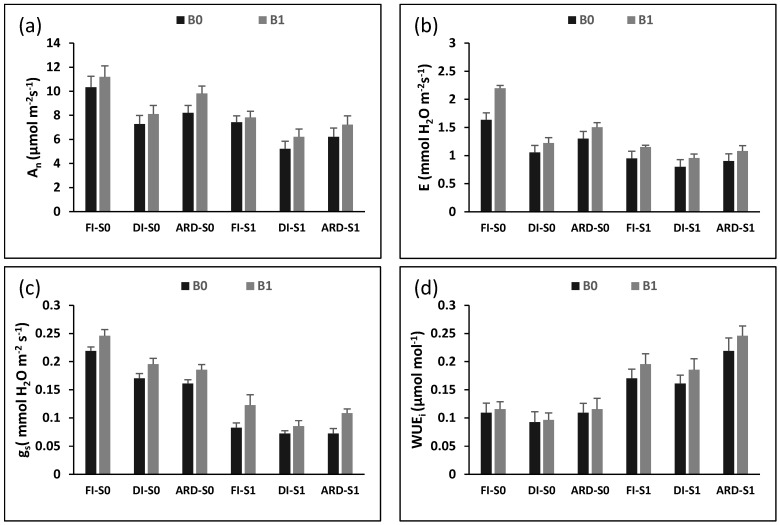
Photosynthetic rate (A_n_) (**a**), transpiration rate (E) (**b**), stomatal conductance (g_s_) (c), and intrinsic water use efficiency (WUE_i_) (**d**) as influenced by biochar (B0 = 0% and B1 = 6%), irrigation regime (FI, DI, and ARD), and NaCl (S0 = 0 mM NaCl and S1 = 300 mM NaCl). Data are means of five replicates *±* SE (*n* = 5). SE denotes standard error.

Figure 1 depicts how Biochar (B), irrigation regime (I), and NaCl (S) affect photosynthetic rate (A_n_), transpiration rate (E), stomatal conductance (g_s_), and intrinsic water use efficiency (WUE_i_). Compared with FI, reduced irrigation (DI and ARD) considerably lowered both A_n_ (*p* = 0.0022; *p* = 0.0015), and g_s_ (*p* = 0.0034; *p* = 0.0020), irrespective of S and B. In all irrigation regimes, biochar addition had a favorable impact on A_n_, E, and g_s_, particularly in ARD with salinity irrigation. While A_n_ was exclusively substantially affected by the S + I interaction (*p* = 0.014), g_s_ showed a prominent significant effect with all interactions (B + I, B + S, S + I) (*p* = 0.0016; *p* = 0.0034; *p* = 0.0022). A_n,_ E, and g_s_ were 14.8%, 33.3%, and 20% greater in B1 plants than in B0 plants for ARD treatment under salinity, respectively. Additionally, the highest WUE_i_ was noticed with the ARD, B1, and saline treatments. Moreover, salinity stress decreased the photosynthetic rate, stomatal conductance, and transpiration rate by 24.2, 46.3.2%, and 30.6%, respectively, when compared with the non-salinity treatment in ARD.

### 2.3. Plant Water Potential and Abscisic Acid Content

**Figure 2:**
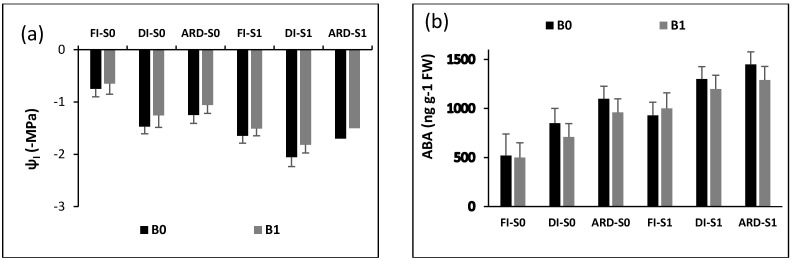
Water potential (ψ_l_) (**a**) and concentration of abscisic acid (ABA) (**b**) of leaves of eggplant variety (‘Bonica F1’) as impacted by biochar doses (B0 = 0% and B1 = 6%), irrigation regimes (FI, DI, and ARD), and NaCl (S0 = 0 mM NaCl and S1 = 300 mM NaCl). Data are means of five replicates *±* SE (*n* = 5). SE denotes standard error.

Figure 2 shows the effects of Biochar (B), irrigation regime (I), and NaCl (S) on water potential (ψ_l_) and abscisic acid (ABA) content. While B, I, S, and S + I had a substantial impact on ψ_l_ (*p* = 0.0012; *p* = 0.0021; *p* = 0.0065; *p* = 0.0011), B, I, S, B + I, and S + I had a significant effect on ABA (*p* = 0.0021; *p* = 0.0013; *p* = 0.0027; *p* = 0.0023; *p* = 0.0026). Limited irrigation significantly raised ABA and lowered water potential (more negative), irrespective of S and B. B, however, significantly improved ψ_l_ and ABA content. Additionally, under the saline condition, B exhibited higher ability in improving ψ_l_ and lowering ABA in ARD in comparison to DI. In contrast, with both normal and saline conditions, biochar hardly affect ψ_l_ and ABA in FI.

### 2.4. Leaf C, and N Content and Chlorophyll Content Index 

**Figure 3:**
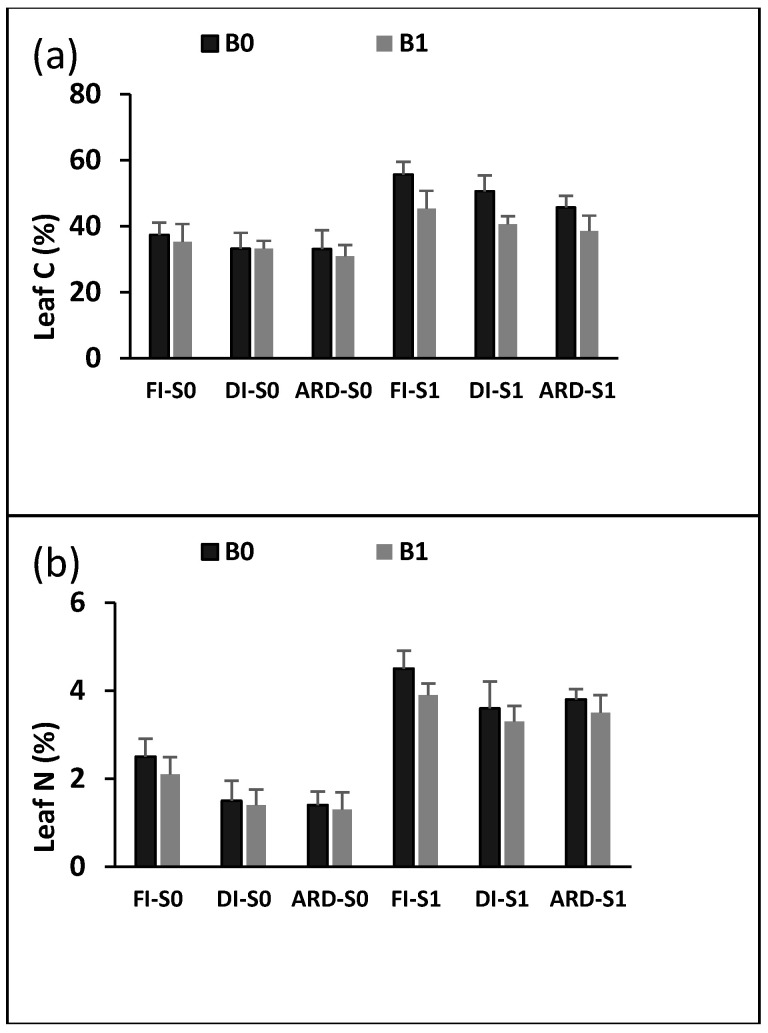

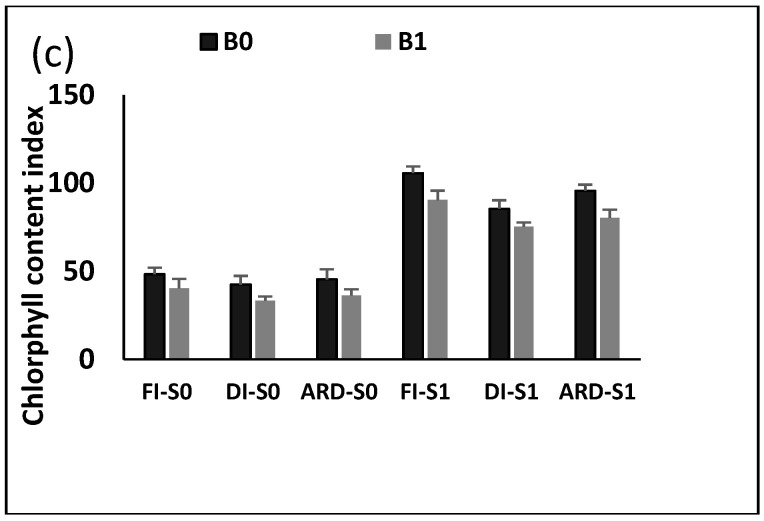
Leaf C (**a**), and N (**b**) concentration and leaf chlorophyll content index (**c**) as influenced by biochar (B0 = 0% and B1 = 6%), irrigation regime (FI, DI, and ARD), and NaCl (S0 = 0 mM NaCl and S1 = 300 mM NaCl). Data are means of five replicates ± SE (*n* = 5). SE denotes standard error.

Salinity hardly affected leaf C concentration (Figure 3c). B, I, S, B + I, and B + S all significantly impacted leaf N concentration (*p* = 0.0029; *p* = 0.0012; *p* = 0.0045; *p* = 0.0021; *p* = 0.0018). Under both normal and salinity conditions, B, I, S, and B had a substantial impact on leaf N concentration (*p* = 0.0023; *p* = 0.0017; *p* = 0.0035; *p* = 0.0041; *p* = 0.0019) (Figure 3b). In the presence or absence of B and S, leaf N and CCI gained higher values under ARD than under DI. B, I, S, B + S, and S + I, all had a substantial impact on the chlorophyll content index (CCI) (*p* = 0.0016; *p* = 0.0022; *p* = 0.0045; *p* = 0.0031; *p* = 0.0025) (Figure 3a). CCI in B0 was higher than B1 in each of the I treatments under saline conditions by 19.2%, 22.5%, and 28.5%, respectively.

### 2.5. Na^+^ and K^+^ Content

**Figure 4:**
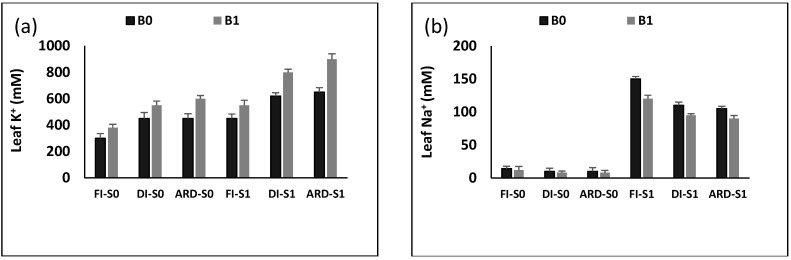
Leaf K^+^ (**a**) and Na^+^ (**b**) content as influenced by biochar (B0 = 0% and B1 = 6%), irrigation regime (FI, DI, and ARD), and NaCl (S0 = 0 mM NaCl and S1 = 300 mM NaCl). Data are means of five replicates *±* SE (*n* = 5). SE denotes standard error.

The impact of Biochar (B), irrigation regime (I), and NaCl (S) on Na^+^ and K^+^ is displayed in Figure 4. B, I, S, B + S, and S + I significantly impacted Na^+^ (*p* = 0.0013; *p* = 0.0032; *p* = 0.0025; *p* = 0.0019; *p* = 0.0028), whereas B, I, S, B + I, and S + I significantly impacted leaf K^+^ (*p* = 0.0033; *p* = 0.0021; *p* = 0.0064; *p* = 0.0042; *p* = 0.0026). Under normal (I) and salinity conditions (S), leaf exhibited higher Na^+^ and lower K^+^ content in FI than those in DI and ARD; however, Na^+^ was lower and leaf K^+^ was higher under combined ARD and B than DI with B. As a result, under salinity, the association of B and ARD improved the K^+^/Na^+^ ratio.

## 3. Discussion

Throughout the whole plant life cycle, salt and drought stresses have a detrimental effect on crop growth, development, and metabolism. Finding practical and efficient approaches to maintain an acceptable level of soil humidity, water availability, and ion uptake in crops under drought and salinity stresses is therefore urgently needed. To deal with drought and salinity stresses, a variety of solutions have been adopted, including water-saving irrigation techniques, the cultivation of tolerant crops, and biochar addition. Further research is required to determine their combined impact on salt and drought stresses. For eggplant’s vegetative and reproductive growth, the flowering stage is a crucial transitional step, and it is vulnerable to salt and drought stress [9,26]. The delay in the eggplant variety (‘Bonica F1’) flowering time observed in this work was caused by salinity and drought stress, which should be the efficient strategy for eggplant adaptation to the challenging habitat. Earlier findings have demonstrated that salt and drought stress can contribute to the delay of plant flowering, which has a detrimental impact on crop yield [9,27,28]. The results of the current study agree with the previous findings, which might be due to higher water usage combined with later flowering times [27].

Biochar improved soil humidity in all irrigation regimes, according to earlier research reported by [29], although it had a greater favorable effect on soil water tenor, particularly under DI (deficit irrigation) and ARD (alternate root-zone drying irrigation) in comparison to FI (full irrigation). Due to its porous nature, biochar addition might reduce soil bulk density and improve soil surface area, thus enhancing the soil’s capacity to absorb and retain water [30].

Furthermore, [31] reported that biochar might improve soil aggregate stability and thus increase soil humidity retention effectively under dry conditions. Likewise, [32] stated that a biochar porous structure boosted up aeration and soil water holding capacity (WHC) while decreasing evapotranspiration. Additionally, according to [33], biochar might increase soil water content, thus diluting ion concentration when exposed to salinity stress and maintaining ideal soil conditions for plant development.

The necessary requirement to guarantee optimal crop growth, particularly under abiotic stress, is a suitable soil water condition. In the current research work, under salt stress, biochar improved eggplant yield in all irrigation regimens. Our results agree with previous findings that have been reported by ref. [34] showing that poultry feather hydrolysate, as a bioenhancer, positively impacted the growth and development of brinjal and chilli plants, along with early flowering and improved crop yield. Similarly, [35] stated that biochar applications alleviated the negative impacts of drought stress on plant growth characteristics of cabbage seedlings.

As stated by ref. [36], abiotic stress significantly affects physiological reactions, thus lowering photosynthetic rate (A_n_) and stomatal conductance (g_s_). Similarly, in the current work, A_n_ and g_s_ considerably declined under restricted irrigation (DI and ARD) in comparison to FI and dramatically plummeted more under salty irrigation, while biochar had a beneficial impact on A_n_ and g_s_ in the presence or absence of salinity. This could be explained by the fact that biochar improves plant water status through the enhancement of soil water content and the adsorption of Na^+^ excess [37,38,39]. The positive effect of biochar use on gas exchange parameters under stressful conditions observed in our results was previously reported by ref. [35], showing that biochar applications improved the leaf water relative content (LWRC), stomatal conductance (g_s_), net photosynthetic rate (Pn), and transpiration rate (Tr) of the cabbage seedlings under water deficit conditions. Enhancement of photosynthetic activity with biochar addition might be explained by the increased soil water holding capacity, porous structure, and high surface area of biochar [35,40].

Besides, the dry zone of ARD induced a lower g_s_ than DI because it caused higher leaf ABA (abscisic acid) buildup than DI. This is in conformity with our results, where a negative correlation was detected between ABA and g_s_ (data not shown). In comparison to DI, ARD showed significantly greater water adjustment capabilities, leading to less negative water potential (ψ_l_). It was evident that under salt stress, the association of biochar and ARD exhibited greater intrinsic water use efficiency (WUE_i_). When subjected to drought and salt stress, plants activate dehydration prevention systems to maintain water status [39,41]. As emphasized by ref. [42], under abiotic stress, ABA functions as a crucial chemical signal that is transmitted between the root and leaf through the xylem flux to manage stomata closure and prevent water dissipation. According to [43], to sustain water intake and cell turgor, plants reduce leaf ψ_l_. In our investigation, biochar-induced elevation in soil accessible water content efficiently enhanced plants water status, which increased leaf water potential (ψ_l_). In contrast to DI, this beneficial effect was considerably magnified with ARD. The raison is that ARD lowered soil ion buildup, which decreased plant absorption of Na^+^ [39,44]. This strategy was also generated by an improved ARD control over the equilibrium of hydraulic and chemical signals. This offered more proof that the ARD irrigation approach outperforms the DI. It is noteworthy that biochar might mitigate osmotic stress by adsorbing excess Na^+^ from soil [39,45]. Likewise, our findings showed that biochar addition, in all irrigation treatments contributed to a decline in Na^+^ uptake and an increase in K^+^ uptake. It is well known that biochar helps reduce salt stress.

In an earlier work, [45] mentioned three potential mechanisms for how biochar can help plants that are stressed by salinity: (i) In the beginning, biochar could have an elevated potential to momentarily bind Na^+^ to its exchanging site, which would lower the balance concentration of Na^+^ in soil solution; (ii) Biochar has a higher moisture-retaining potential, thus diluting salt and reducing osmotic stress; (iii) biochar may boost up soil mineral concentration by providing specifically K^+^, Ca^2+^, and Mg^2+^, which could have an impact on lowering plant absorption of Na^+^. In the present investigation, we also discovered that biochar significantly reduced the stress caused by salinity on eggplant under normal and limited irrigation treatments.

Along the nitrogen cycle of soil, including nitrification and plant N absorption, biochar also plays a key role. In an earlier study, it was reported that biochar may reduce nitrification and boost ammonia absorption [46]. Moreover, ref. [47] stated that biochar might improve soil’s ability to store ammonium and lessen nitrate leaching. In our situation, leaf C and N concentration decreased under restricted hydric conditions (DI and ARD) compared with FI and rose when exposed to salty conditions, whereas biochar clearly had a detrimental impact on leaf N uptake under all irrigation treatments. Due to its high C/N ratio, biochar may induce soil N immobilization and reduce the amount of N that is available [48,49]. As emphasized by ref. [50] nutrient immobilization was also generated by using a low amount of biochar. However, the impact of biochar on crops’ ability to absorb nitrogen needs to be further investigated. Although biochar addition has a high C/N ratio, organic C in biochar is hard to mineralize, so N immobilization may not be very significant. Both the particle size of biochar and the feedstock types may have an impact on plant N buildup and potentially have the reverse effect, according to ref. [51]. In the current work, the chlorophyll content index (CCI) was also decreased, along with a reduction in leaf N. Our finding is in concordance with earlier research, showing that the chlorophyll content index (CCI) is a crucial indicator that may reflect the crop nitrogen nutrition potential [29].

Despite the decrease in leaf N content decrease, we found a significantly higher yield with biochar additions. Regarding N uptake in the presence or absence of salinity, ARD outperforms DI. This strategy is induced by combining ARD and biochar. It is worth noting that biochar might effectively boost soil fertility and improve plant nutrient absorption [52]. Our results are consistent with previous findings showing that ARD and biochar preserved crop water intake and ameliorated plant physiology [29].

## 4. Materials and Methods

### 4.1. Biochar Description

Biochar was produced from air-dried maize straw via a gradual pyrolysis procedure that involves heating maize straw in an oxygen-free environment. The maize straw was heated throughout this process to a maximum temperature of 550 °C. The Laboratory of Biotechnology and Plant Physiology (National Institute of Agricultural Research) provided the maize straw, which was air dried for during 48 h at 35 to 40 °C. After being air dried, the maize straw was placed in aluminum bags of 30 × 10 × 10 cm (3 L volume) and pyrolyzed under oxygen-limited conditions in an electrical furnace. To produce a fully carbonized biochar with a high fixed carbon content, the temperature of the pyrolysis process was constantly raised at a rate of 5 °C min^−1^ up to a maximum of 550 °C. After three hours of heating, the biochar was cooled overnight to room temperature. The biochar was then mixed. The main properties of biochar were 72.4% of Carbon, 1.62% of Nitrogen, 1.72% of Hydrogen, 30.2% of ash content, 13.0 cmol (+)/kg of Cation exchange capacity, 1.07 of Electrical conductivity and a pH of 10.1.

### 4.2. Pot Experiment

A pot experiment was carried out in climate chambers at the Laboratory of Biotechnology and Plant Physiology of the National Institute of Agricultural Research (36°50′ N, 10°11′ E), Tunis, Tunisia. The following settings were made for climate parameters in two growth chambers: Temperature: 25/21 ± 1 °C at day/night; relative humidity: 60 ± 2%; during 13-h photoperiod, light was provided by lamps, with the light magnitude averaging from 570 to 670 µmol m^−2^ s^−1^. The plant material used in our experiment was *Solanum melongena*, cv. ‘Bonica F1’, which is cultivated very frequently and is therefore of economic importance. Seeds of ‘Bonica F1’ (Vilmorin, France) provided by the Biology Department (College of Science, University of Hail), were grown in climate-controlled chambers in plastic trays filled with peat. Twenty-five days later, one seedling was transferred into plastic pots with a height of 10 cm and a diameter of 4 cm that contained 4.5 kg of sandy loam soil. Before adding soil to the pots, biochar was incorporated at a level of 6% by weight. Pots without biochar were used as controls. The following nutrients were added to the soil: 80 g N, 30 g P, 90 g K, and 9 g Mg kg^−1^ soil. In the absence or presence of salinity (S0 = 0 mM of NaCl and S1 = 300 mM of NaCl), the plants were treated with three irrigation regimes: full irrigation (FI), deficit irrigation (DI), alternate root-zone drying irrigation (ARD), and one biochar dose (B1 = 6% *w*/*w* and control = B0 = 0% *w*/*w*). Each treatment was applied to 20 plants. The experiment was designed as a randomized complete block design, with four blocks. Each experimental unit contained five plants.

### 4.3. Irrigation Treatments

Following a week after transplantation, the salt-stressed plants were irrigated with 300 mM NaCl solution, while the control plants received S0 = 0 mM NaCl solution (distilled water). The concentration of NaCl was raised stepwise by 80 mM NaCl until it reached S1 = 300 mM NaCl. In the meantime, three irrigation regimes (FI, DI, and ARD) were applied to the plants, namely: (1) plants were watered under the FI regime in the entire pot to the soil’s water-holding capacity (30% of the volumetric water content of the soil). (2) In the ARD treatment, pots were divided into two equal portions in a vertical manner, and water was provided to one portion of the soil’s water holding capacity at a time. When the soil moisture level dropped to 10%, irrigation was shifted to the drier section. (3) Plants were watered evenly throughout the container in the ARD regime but not in the DI regime. Ref. [53] description of how to estimate water holding capacity (WHC) was followed. Utilizing oven drying, the soil moisture in pots was assessed [54].

### 4.4. Phylogeny, Yield and Growth Parameters

On 20 plants, the evaluation of phylogeny (flowering time: FT in days), yield, and growth characteristics was achieved. Forty-five days after flowering, the fruits were picked, numbered, and promptly weighed. The number of fruits per plant (FN) and the mean fresh weight per fruit (FW) (g) were then determined. Plant height (PH) (cm) was gauged at harvest. After being dried in the oven at 70 °C until it reached a consistent weight, the aerial biomass (AB) (g) in the plant’s aboveground parts (shoots and leaves) was collected and determined.

### 4.5. Physiological Parameters and Water Status Measurements

The upper canopy of completely grown leaves was examined for photosynthetic rate (A_n_) and stomatal conductance (g_s_) by using a portable photosynthetic system (LiCor-6400, LI-Cor Bioscience, NE, USA). The measurements were carried out on a single leaf per pot at a CO_2_ content of 400 ppm, a photon flux density of 1200 mol m^−2^ s^−1^, and a chamber temperature of 28.5 °C. WUE_i_ (intrinsic water use efficiency) was estimated using the A_n_/g_s_ ratio. A portable leaf chlorophyll meter (CCM-200; Opti-Science, Tyngsboro, MA, USA) was used to measure the Chlorophyll Content Index (CCI).

The same leaf was immediately detached after the gas exchange measurement for determination of the water potential using a pressure chamber (3000 Series Plant Water Status Consoles, Soil Moisture Equipment Corp., Santa Barbara, CA, USA). The leaf water potential was expressed in–Mpa.

### 4.6. Leaf Abscisic Acid (ABA), Nitrogen, Carbon, Na^+^, and K^+^ Content

Using a mortar and pestle, 30 mg of leaf samples were crushed with liquid nitrogen and 1 mL of milli-Q H_2_O. After that, samples were carried and homogenized in Eppendorf tubes by shaking for 24 h at 4 °C, followed by 10,000× *g* centrifugation during a 5 min at 4 °C. Until analysis, the clear supernatant was gathered and kept at 4 °C. A monoclonal ABA antibody (AFRC MAC252) was used following the ELISA method to measure the ABA concentration in leaf samples [55]. The leaf ABA concentration was expressed in ng g^−1^ FW.

Using an Elemental Analyzer System, the nitrogen and carbon concentrations (%DW) of finely powdered dry leaf samples were measured (vario PYRO cube, Elementar Analyzer system GmbH, Germany).

HNO_3_ was used to digest the ground-up dry leaf samples. We used the atomic absorption spectrometer (ICE_3500_, Thermo Fisher Scientific Inc., Waltham, MA, USA) to measure the concentrations of Na^+^ and K^+^ (mM).

### 4.7. Statistical Analysis

All analyses were performed using a completely random design. After running a three-way analysis of variance on all the obtained data, SPSS Statistics 25 (IBM SPSS Statistics) was used to find significant differences between the treatments (ANOVA). The Tukey multiple range test was used to compare the means (*p* = 0.05). To ascertain the correlation between some of the variables, regression analyses were utilized.

## 5. Conclusions

Biochar may improve plant water status by raising the soil water content. Moreover, under salt stress, biochar may absorb more Na^+^. Compared with DI, these beneficial effects were increased even more under ARD. ARD exhibited much better water adjustment potential than DI. Eggplant growth was enhanced using biochar in association with lowered water supply treatments, notably ARD under salty conditions. When applying biochar to soil, consideration should be given to its quantity and characteristics. While ABA production was boosted as a result of the ARD and biochar combination, water availability and ionic equilibrium were also preserved, ensuring improved WUE_i_ and yield parameters for plants subjected to salt stress. Even though biochar had obvious adverse impacts on the absorption of nitrogen, it might improve nutrient supply and plant nutrient absorption. To alleviate NaCl stress, enhance water use efficiency, and improve plant yield under drought stress when cultivating the salt-tolerant eggplant cultivar ‘Bonica F1’, the adoption of the combination of ARD and biochar addition may be an efficient and successful strategy. To ensure sustainable agriculture in the future, we should consider the interactions between various abiotic limiting factors on crop development. From a more fundamental perspective, the expansion of the range of tested crop varieties and the investigation of field conditions could be of interest in the future.

## Figures and Tables

**Table 1:** Effect of biochar (B0 = 0% and B1 = 6%), irrigation regime (FI, DI, and ARD), and NaCl (S0 = 0 mM NaCl and S1 = 300 mM NaCl) on eggplant variety (‘Bonica F1). The variable tested were: flowering time (FT), plant height (PH), aerial biomass (AB), fruit per plant (N), and mean fresh weight per fruit (FW).

Treatments			Flowering Time (FT) (Days)	Plant Height (PH) (cm)	Aerial Biomass (AB) (g)	Fruit per Plant (N)	Mean Fresh Weight per Fruit (FW) (g)
S0	FI	B0	46.75 ± 0.32 g	11.4 ± 0.5 b	120.3 ± 1.2 b	8 ± 0.1 b	180 ± 1.9 b
		B1	43.5 ± 0.42 h	13.3 ± 0.2 a	150.1 ± 3.5 a	10 ± 0.2 a	195 ± 3.6 a
	DI	B0	52.34 ± 0.26 e	7.1 ± 0.1 d	70.5 ± 2.1 f	5 ± 0.4 d	140 ± 2.5 d
		B1	49.36 ± 0.41 f	8.5 ± 0.4 c	85.3 ± 5.2 f	6 ± 0.6 c	150 ± 1.9 c
	ARD	B0	51.02 ± 0.31 e	9.4 ± 0.2 c	95.2 ± 3.2 e	6 ± 0.1 d	147 ± 4.1 d
		B1	49.96 ± 0.41 f	11.1 ± 0.3 b	110.1 ± 1.7 d	7 ± 0.3 c	155 ± 2.9 c
S1	FI	B0	55.56 ± 0.32 d	6.5 ± 0.3 f	100.2 ± 4.1 d	3 ± 0.2 e	70 ± 1.3 e
		B1	54.10 ± 0.40 d	7.6 ± 0.2 e	118.3 ± 3.3 c	3.5 ± 0.2 e	75 ± 5.0 e
	DI	B0	63.08 ± 0.42 a	5.3 ± 0.5 h	45.7 ± 1.5 h	1.5 ± 0.3 h	35 ± 2.2 h
		B1	60.54 ± 0.26 b	5.7 ± 0.6 g	55.8 ± 2.0 h	1.8 ± 0.1 g	40 ± 3.1 g
	ARD	B0	59.17 ± 0.40 b	5.8 ± 0.4 g	67.8 ± 3.9 g	1.9 ± 0.2 g	45 ± 2.0 g
		B1	56.12 ± 0.30 c	6.5 ± 0.2 f	75.9 ± 1.7 f	2.4 ± 0.2 f	55 ± 1.9 f

Data are means *±* SE of five repetitions. Different lowercase letters denote significant differences between treatments (*p* ≤ 0.05) based on Tukey’s HSD test.

## Data Availability

No new data were created or analyzed in this study. Data sharing is not applicable to this article.
